# Comparing Intellectual and Memory Abilities of Older Autistic Adults with Typically Developing Older Adults Using WAIS-IV and WMS-IV

**DOI:** 10.1007/s10803-019-04122-w

**Published:** 2019-08-22

**Authors:** Venus W. S. Tse, Jason Crabtree, Shamsun Islam, Joshua Stott

**Affiliations:** 1 Research Department of Clinical, Educational and Health Psychology, University College London UCL, Gower Street, London, WC1E 6BT UK; 2 East London NHS Foundation Trust, London, UK; 3 North East London Foundation Trust, London, UK

**Keywords:** Autism, Older adults, Memory, Cognitive, Processing speed

## Abstract

This study aimed to compare cognitive and memory abilities between older adults with and without autism over the age of 50. Twenty-eight individuals with autism and 29 typically developing (TD) older adults took part in the current study. Participants’ cognitive and memory abilities were assessed by WAIS-IV and WMS-IV. Older autistic adults were found to have poorer performance in processing speed and visual working memory, but they performed at a similar level as TD controls in all other domains. Poorer processing speed and visual working memory are also often found to be associated with age-related decline in neurotypical adults. Longitudinal studies are warranted to explore how the combination of ageing and autism affects cognitive functioning in older adults.

## Introduction

Ageing in typically developed (TD) adults is associated with decline in cognition, with specific subtypes of memory (McDaniel and Einstein 2011) and specific aspects of overall cognitive functioning, such as processing speed, affected (Grady 2012). There are a number of studies pointing to differences in executive function and episodic memory between children and young adults with Autism and their TD peers (Barendse et al. 2013; Boucher et al. 2012; Geurts et al. 2009; Geurts and Vissers 2012; Happe et al. 2006; Hill 2004; Pellicano 2010; Pennington and Ozonoff 1996; Sachse et al. 2013; Sergeant et al. 2002). However, Autism is a lifelong condition (Brugha et al. 2011) and it remains unclear whether or how having Autism affects degree or type of cognitive change in ageing (Lever and Geurts 2016). Understanding differences between people with an Autism diagnosis and TD adults as they age is of clinical importance as significant decline in specific areas of cognitive functioning can be a prodromal indication of dementia (Albert et al. 2011). For example, the most common form of dementia, Alzheimer’s disease, starts with initial episodic (particularly delayed memory) impairment (Salmon and Bondi 2009).

Work in younger adults with Autism is informative, but cannot be directly transferred, given the potential change in cognition as individuals age (Lever and Geurts 2016). Work in younger adults with Autism has indicated that they differ in specific aspects of intellectual functioning from TD adults even when their overall intellectual functioning is in the average range. For example, verbal IQ is, on average, similar to TD adults, but processing speed (Geurts et al. 2008; Mayes and Calhoun 2008; Oliveras-Rentas et al. 2012) and working memory (Barendse et al. 2013) tend to be poorer.

Research in older adults with Autism is very scarce compared to research in children and adolescents with Autism. This is perhaps because it has been difficult to recruit older adults with a confirmed diagnosis of Autism since Autism was only included in the psychiatric classification system about three decades ago (van Niekerk et al. 2011). There has been a lack of research in psychosocial and cognitive outcomes in adults aged over 50 with Autism and thus there is an urgent need to address this gap in the literature so as to understand the unique needs of older adults with Autism and also how best to support them. Several studies examined memory abilities in older adults with Autism but findings were not consistent (Davids et al. 2016; Geurts and Vissers 2012; Lever and Geurts 2016). For example, one study suggested that older people with Autism and average or above IQ have a similar pattern of memory strengths and weaknesses to that found in younger adults with Autism and average IQ and concluded that in some cases weaknesses may improve with age relative to TD adults (Lever et al. 2015). By contrast, a second study tentatively concluded that age and a diagnosis of Autism may interact leading to greater cognitive decline relative to TD adults in visual memory (Geurts and Vissers 2012).

These inconsistent findings as to memory may, in part, be due to lack of reliability and validity of assessment tools. Studies thus far (Geurts and Vissers 2012; Lever and Geurts 2016; Lever et al. 2015) have used single subtests from different assessment batteries to tap particular aspects of memory. This may lack both reliability and content validity (Crawford et al. 2012). Thus, there is a need to examine memory profiles using reliable and well-validated assessment batteries that employ multiple subtests to assess a broad spectrum of memory functions, based on a theoretically consistent model. One such assessment battery is the Wechsler Memory Scale—Fourth Edition (WMS-IV; Wechsler 2009a).

Spek et al. (2017) compared cognitive profiles between older adults with and without Autism with the use of Wechsler Adult Intelligence Scale-Third Edition (WAIS-III; Wechsler 2008a). They found that the two groups only differed in processing speed whereby TD controls outperformed older adults with Autism. Among the two processing speed subtests, a significant difference only emerged in Digit Symbol coding (DSC) but not Symbol Search (SS), with the TD controls outperforming older adults with Autism in the DSC subtest. While Spek et al. (2017) used the WAIS-III, the newer edition the WAIS-IV has been found to be a better measurement of intelligence as it provides a better measure of working memory and processing speed (Taub and Benson 2013). Given that individuals with Autism tend to have poorer performance in both of these areas, the WAIS-IV seems to be a better measure to employ so as to further our understanding of the cognitive profile in older adults with Autism. Furthermore, since the WAIS-IV was co-normed with the WMS-IV, its inclusion in the current study allowed for adjustment of each individual’s memory scores for their intellectual abilities. This facilitated interpretation of any obtained memory differences between TD and autistic groups as any effect of IQ differences was obviated. In general, understanding the nature of memory and intellectual ability profiles in older adults with Autism may inform the use of neuropsychological assessment in dementia diagnostic settings (Salmon and Bondi 2009), since understanding what is typical in those with Autism will help inform understanding of whether cognitive decline is present or not (Geurts and Vissers 2012).

In summary, the current study aimed to address a gap in the literature by measuring profiles of intellectual abilities in adults aged over 50 with and without Autism using a reliable and well validated measure. Another aim was to add to the limited previous work examining memory differences in these groups by using a theoretically derived, well-validated measure of memory to further elucidate memory profile differences between adults aged over 50 with and without Autism. The over 50 age cut off was used based on previous work in this area (Geurts and Vissers 2012; Lever and Geurts 2016) and a recent policy report on aging with Autism by the National Autistic Society (2013) which defined ‘older’ as over 50 years of age. In the light of conflicting findings from previous research, this study aimed to address the following questions:

1. Are there significant differences between people aged over 50 with and without Autism on indices of the WAIS-IV? It is hypothesised that similar to younger people with Autism and results from Spek et al.’s (2017) study, older adults with Autism will have lower scores in processing speed and working memory indices relative to TD controls.
2. Are there significant differences between people aged over 50 with and without Autism in WMS-IV memory indices when: (a) memory index scores are adjusted for IQ and (b) memory index scores are not adjusted for IQ?

## Method

### Design

A between groups cross sectional design was used.

### Sample Size

Power analysis for this study was informed by the study conducted by Geurts and Vissers (2012), which compared cognitive abilities between individuals with and without Autism over 50 years of age. Their results suggested that older adults with Autism exhibited deficits in working memory with a large effect size. Calculations using G*Power (Faul et al. 2007), suggested that the sample size required to find a similar effect size, (where a between groups *t* test was used with power (1-beta) = 0.8 and alpha = 0.05) would be 21 participants in each group. To be conservative, we aimed to over-recruit by a third.

### Ethical Approval

The present study was approved by the UCL Research Ethics Committee [11161/001].

### Participants

#### Autism Group

A convenience sample of 28 adults aged over 50 with Autism were recruited from four sources: (i) participants agreeing to be contacted following a previous study (Hickey et al. 2017); (ii) an Autism register held at UCL; (iii) community based groups and (iv) online advertisement. Inclusion criteria included fluent English speakers aged 50 years old or above with Autism, defined here as a diagnosis of Autism Spectrum Disorder (ASD) (Diagnostic and Statistical Manual of Mental Disorders—Fifth Edition (DSM-5), American Psychiatric Association 2013a, b) or, as many diagnoses preceded the DSM-5 criteria, i.e. Autistic disorder, Asperger Syndrome (AS) and Pervasive Developmental Disorder Not Otherwise Specified (*PDD*-*NOS*) (Diagnostic and Statistical Manual of Mental Disorders—Third Edition (DSM-III), American Psychiatric Association 1980; National Autistic Society 2016). Diagnostic status of all individuals with Autism was based on self-report, with participants providing their diagnosis and the name of the service in which they received their diagnosis. Participants were excluded from the present study if they reported to have any current or history of other neurodevelopmental disorders (i.e. ADHD) or cognitive difficulties, a diagnosis of learning difficulties/disability (or IQ < 70 on the WAIS-IV used in this study) or a sensory disability (uncorrected severe visual impairment) that would prevent them from engaging with the test material.

#### Control Group

An IQ matched sample of 29 TD individuals was recruited for this study through community groups and advertising on online platforms. All participants were fluent English speakers aged 50 years old or above and had an IQ of 70 or above. None of the 29 control participants reported any current or history of any cognitive difficulties, global or specific learning or sensory disability that would prevent them from engaging easily with the test material. All control participants completed the Adult Autism Spectrum Quotient (AQ; Baron-Cohen et al. 2001) to screen for Autism. Participants who scored above cut-off (≥ 32) on the AQ, were excluded from the study.

### Materials

#### Demographic Information

Participants’ demographic information was collected by a demographic questionnaire that was designed for the present study.

#### Autism-Spectrum Quotient (AQ)

AQ (Baron-Cohen et al. 2001) is a 50 item self-report measure of autistic traits (attention switching, communication, social skills, attention to detail and imagination) with good test–retest reliability (Baron-Cohen et al. 2001, 2006; Hoekstra et al. 2008). There are four response options ranging from definitely disagree to definitely agree with a response endorsing symptoms of Autism receiving a score of 1. Total scores range from 0 to 50, with higher scores indicating higher levels of autistic traits. The clinical cut-off of AQ is ≥ 32 and screening cut-off value is ≥ 16 (Baron-Cohen et al. 2001). AQ has been replicated across different ages (Auyeung et al. 2008) and cross-culturally (Hoekstra et al. 2008; Wakabayashi et al. 2006). In the current study AQ was only given to the controls for exclusion criterion purposes as detailed earlier.

#### Wechsler Adult Intelligence Scale—Fourth Edition (WAIS-IV)

WAIS-IV (Wechsler 2008a) is a reliable measure of intellectual abilities, with large representative normative data and extensive research indicating its high levels of validity and reliability to assess cognitive ability in individuals aged 16–90 (Climie and Rostad 2011). The WAIS-IV has 15 subtests, 10 of which are ‘core’ and were completed in the current study. These 10 tests are scored on a scale from 1 to 19 with a mean of 10 and standard deviation of 3. The scaled scores are combined to create six composite score indices (range 50–150, Md = 100, SD = 15) derived from theoretical and factor analytic models (Wechsler 2008b, c). Indices include: (i) Verbal Comprehension Index (VCI), (ii) Perceptual Reasoning Index (PRI), (iii) Working Memory Index (WMI), (iv) Processing Speed Index (PSI), (v) General Ability Index (GAI), and (vi) Full-Scale IQ (FSIQ). Both GAI and FSIQ provide an overall estimate of an individual’s intellectual ability, but they differ in how they are derived. FSIQ is derived from the 10 cores subtests, whereas GAI is derived from only core Verbal Comprehension and Perceptual Reasoning subtests. Compared to FSIQ, GAI provides an estimate of general intellectual ability that has a reduced emphasis on working memory and processing speed. The dependent variables used to examine intellectual ability profile in the current study were VCI, PRI, WMI and PSI.

#### Wechsler Memory Scale—Fourth Edition (WMS-V)

WMS-IV (Wechsler 2009a) is a reliable and well-validated measure of memory abilities in individuals aged 16–90 (Horne and McDonald 2012). It consists of ten subtests, which contribute to five primary index scores derived from factor analytic work and theoretical models of memory (Wechsler 2009b, c). Indices include: Auditory Memory Index (AMI), Visual Memory Index (VMI), Visual Working Memory Index (VWMI), Immediate Memory Index (IMI) and Delayed Memory Index (DMI).

IQ adjusted Memory Index Scores can be obtained in the form of Contrast Scaled Scores (CSS). These represent the examinee’s rank order on memory indices relative to intellectual functioning equivalent peers in the WMS-IV standardisation sample. Consequently, a CSS is a measure of the level of performance on an index in comparison to general ability matched peers. A CSS for each memory index can be obtained from a table in the WMS-IV manual using a participant’s obtained WAIS-IV GAI and WMS memory index scores. CSS range from 1 to 19 and have a mean of 10 and a standard deviation of 3. In the current study original indices were used in the primary analysis with a sensitivity analysis run to see if results held true when CSS were used.

### Procedure

Participants were first asked to read the information sheet of the present study and then they were asked to complete a consent form and a demographic questionnaire. The WAIS-IV and WMS-IV were then administered. To minimize order effects, the order in which WMS-IV and WAIS-IV were administered was counterbalanced across participants. Participants in the control group also completed the AQ to assess the presence of any autistic traits. Following successful completion of the testing session, all participants were sent a brief report about their assessed performance.

### Data Analysis

All data were analysed using SPSS version 25. Participants’ characteristics were compared to see if there were any significant differences between the two groups apart from Autism status. Chi Square tests or Fisher’s exact tests were used for categorical data. Continuous data were evaluated as to whether they met assumptions of parametric statistical tests. Depending on whether data (transformed or otherwise) met parametric assumptions, independent sample t-tests or Mann–Whitney U tests were used to compare the participant characteristics and different index score between Autism and control groups. Due to the multiple comparisons made between groups across different cognitive domains, Bonferroni corrected *p*-values were employed to control for Type I error.

As observed index score differences between TD and Autism groups might be due to demographic differences between these groups rather than Autism per se, it was considered important to test whether group differences in index scores remained after accounting for demographic differences. Consequently, exploratory hierarchical regression analyses were conducted for each IQ/memory index score that differed significantly between TD and Autism groups. The dependent variable in each of these regressions was an index score that differed significantly between the two groups and the independent variables were Autism status and any demographic variable that: (a) significantly differed between TD and Autism groups, and (b) correlated significantly with index score performance. Demographic variables were entered as a first step in the regression. Autism status was then entered as a second step to assess whether having Autism still had an influence on index score performance over and above any important demographic difference between the TD and Autistic groups.

## Results

### Participant Characteristics

In total, 119 individuals emailed the researcher to express interest in participating in the study. At the end, 57 individuals consented to take part in the current study, 29 of them were TD older adults and 28 were older adults with Autism. Two TD controls were excluded from the analysis as their AQ ≥ 32 leaving 27 TD adults in the final analyses. Table 1 summarizes participants’ characteristics and differences in demographics between the controls and Autism group. The groups did not differ significantly from one another in age or overall IQ. In addition, no significant group difference was found in ethnicity (White/non-white) or level of education. However, there was a significant difference in gender between Autism and control group, *x*^2^(1) = 11.44, *p *= 0.01.

**Table 1:** Characteristics of participants with autism and matched controls

	Group	
	Autism (*n *= 28)	Control (*n *= 27)
Age^a,^^		
Median^b^ (range)	61 (50–72)	63 (50–68)
Gender*^,c^	22 M/6 F	9 M/18 F
IQ^d,^^		
Mean (SD)	113.39 (18.11)	118.89 (11.59)
Ethnicity^e,f,^^		
White/non-White	25/3	23/4
Education^c,g,^^		
No higher education/higher education	7/21	7/20
AQ		
Mean (SD)	–	17.33 (6.20)

^a^Mann–Whitney U-test was used

^b^Median was reported due to non-normal data distribution, Mann–Whitney U-test was used as a significance test

^c^Chi square test was used

^d^Independent-sample t-test was used

^e^The number between slash indicates number of participants who are white/who are non-white

^f^Fisher’s exact test was used

^g^The number between slash indicates number of participants who did/did not receive higher education. According to the National Qualification Framework (NQF), individuals who achieved NQF level 4 (i.e. above A-levels) are considered as receiving higher education and the present study also adopted the same framework in assessing whether participants had higher education

*p = 0.05

^No significant group differences were found

### Differences in Intellectual Ability Indices Between TD Over 50 s and Over 50 s with Autism

Table 2 summarises participants’ WAIS-IV index scores across different cognitive domains. Four t-tests were conducted and thus the Bonferroni corrected *p*-value is 0.0125. There was a statistically significant difference in PSI scores between the two groups, with control group (*M *= 111.30, *SD* = 13.12) scoring higher than the Autism group (*M *= 99.39, *SD* = 15.33), *t*(53) = 3.09, *p *= 0.003, *d* = 0.91. The only domain that the Autism group had a higher mean index score than the controls was VCI, but the difference was not found to be significant and the effect size of the difference was small (*d* = 0.34). There were no statistically significant differences between groups in other domains.

**Table 2:** Group comparisons on each WAIS-IV Index Score

	Control *n* = 27	Autism *n* = 28	*p*-value	Effect size (Cohen’s *d*)	Total sample *N *= 55
WAIS-IV Index Scores, Mean (SD)					
VCI	112.74 (11.27)	116.54 (15.47)	0.31	0.34	114.67 (13.59)
PRI	118.74 (13.96)	111.14 (18.08)	0.09	0.54	114.87 (16.49)
WMI^a^	116.85 (11.33)	114.25 (16.87)	0.51	0.23	115.53 (14.35)
PSI	111.30 (13.12)	99.39 (15.33)	0.003*	0.91	105.24 (15.38)

^a^The scores were winsorized due to extreme outliers

*p < 0.0125 indicating significant *p*-value after Bonferroni correction

Follow-up analyses (t-tests) were performed to explore if there was a significant difference between two groups in the two PSI subtests. TD controls outperformed those with Autism on both PSI subtests (SS: *t* (53) = 2.48, *p *= 0.02; DSC: *t*(53) = 3.46, *p *= 0.001).

### Differences in Memory Indices Between TD Over 50 s and Over 50 s with Autism

Five group comparisons (all t-tests) were conducted and thus the Bonferroni corrected *p*-value is 0.01. Table 3 summarises participants’ WMS-IV index scores across different domains. There was a statistically significant difference in VWMI between the two groups, with control group (*M* = 116.40, *SD* = 14.53) scoring higher than the Autism group (*M* = 102.86, *SD* = 18.24), *t* (53) = 3.04, *p *= 0.004, *d *= 0.93.

**Table 3:** Group comparison on each WMS-IV index score

	Control *n* = 27	Autism *n* = 28	*p*-value	Effect Size (Cohen’s *d*)	Total sample *N *= 55
WMS-IV Index Scores, Mean (SD)					
AMI	114.59 (15.56)	108.96 (18.02)	0.22	0.36	111.73 (16.94)
VMI^a^	106.85 (18.08)	98.29 (14.45)	0.06	0.47	102.49 (16.74)
VWMI^a^	116.40 (14.53)	102.86 (18.24)	< 0.01*	0.93	109.51 (17.74)
IMI	111.63 (16.47)	104.00 (17.23)	0.10	0.46	107.75 (17.15)
DMI	113.41 (16.17)	105.11 (18.68)	0.08	0.51	109.18 (17.83)

^a^The scores were winsorized due to extreme outliers

*p < 0.01 indicating significant *p*-value after Bonferroni correction

### Differences in IQ Adjusted Memory Indices (CSS) Between TD Over 50 s and Over 50 s with Autism

Table 4 summarises participants’ CSS across different memory domains. In total, five group comparisons (one t-test and four Mann–Whitney U-tests) were conducted and thus the Bonferroni corrected *p*-value is 0.01. As with non-adjusted memory index scores, there was a statistically significant difference found in the CSS of VWMI between the two groups, in which the control group (*Mdn *= 12.00) performed better than the Autism group (*Mdn *= 8.00), *U* = 190.00, *z* = − 3.18, *p *< 0.01, *r* = 0.43.

**Table 4:** Group comparison on each WMS-IV Contrast Scaled Score

	Control *n *= 27	Autism *n* = 28	*p*-value	Effect size	Total sample *N *= 55
WMS-IV Contrast Scale Scores					
AMI^a^					
Mean (SD)	12.26 (3.12)	10.86 (3.90)	0.15	0.45^c^	11.55 (3.58)
VMI^b^					
Median (range)	10.00 (4.00–17.00)	8.00 (3.00–13.00)	0.10	0.22^d^	8.00 (3.00–17.00)
VWMI^b^					
Median (range)	12.00 (6.00–16.00)	8.00 (1.00–17.00)	< 0.01*	0.43^d^	10.00 (1.00–17.00)
IMI^b^					
Median (range)	11.00 (5.00–16.00)	9.5 (1.00–16.00)	0.19	0.18^d^	10.00 (1.00–16.00)
DMI^b^					
Median (range)	11.00 (6.00–18.00)	9.00 (2.00–19.00)	0.06	0.25^d^	10.00 (2.00–19.00)

^a^Independent-samples t-test was used to compare the mean index scores between Autism and control group

^b^Mann–Whitney U test was employed to compare the medians of index scores between Autism and control group

^c^Effect size was calculated in the form of Cohen’s *d*, where *d *= 0.2 (small effect); *d *= 0.5 (medium effect) and *d *= 0.8 (large effect size)

^d^Effect size was calculated in the form of Pearson’s correlation coefficient (*r*), where *r* = 0.10 (small effect); *r *= 0.30 (medium effect) and *r *= 0.50 (large effect)

*p < 0.01, indicating significant *p*-value after Bonferroni correction

### Exploratory Regression

For the two significant differences between groups (the PSI of the WAIS-IV and the VWMI of the WMS-IV), it was assessed whether any demographic differences between groups might confound the difference in the mean index scores of the two groups. For PSI, the only demographic variable associated with performance was gender (*r* = 0.28, *p *= 0.04). For VWMI, no demographic variables were associated with performance. Consequently, one hierarchical multiple linear regression was performed with gender entered first as a predictor of PSI performance and diagnosis entered afterwards. The full model of gender and diagnosis to predict PSI scores was statistically significant, *R*^2^ = 0.17, *F* (2,52) = 5.18, *p *< 0.01; adjusted *R*^2^ = 0.13. Results indicated that having an Autism diagnosis was associated with reduced PSI over and above gender. Specifically, the addition of diagnosis to the prediction of PSI scores led to a statistically significant increase in *R*^2^ of 0.09, *F* (1,52) = 5.40, *p *< 0.05. In addition, diagnosis was a significant predictor of performance (*β *= − 0.33, *p *= 0.02) with an Autism diagnosis associated with 0.33 point decrease in PSI. Gender was no longer a significant predictor when diagnosis was added to the model.

## Discussion

This was the first study to examine intellectual ability and memory ability differences between older autistic adults and TD older adults using reliable and well-validated measures (WAIS-IV and WMS-IV).

### Cognitive Abilities

Over 50s with Autism were found to be performing at a similar level as those without Autism on verbal comprehension, perceptual reasoning and working memory. However, at the same time, they also had significantly lower scores on the processing speed index compared to the controls. This finding is in keeping with results reported by Spek et al. (2017) who also found that older adults with Autism performed significantly worse than TD older adults on processing speed measures. Impairment in processing speed has also been found in previous studies with children and adults with Autism (Geurts et al. 2008; Powell et al. 2017; Spek et al. 2008). Deficits in processing speed refer to difficulties in processing and integrating visual information and then responding to this information. Poor performance in processing speed in individuals with Autism might be due to weak central coherence and differences in executive functioning, both of which are theorised to be characteristics of individuals with Autism. With respect to weak Central Coherence, processing speed performance of individuals with Autism might be affected by focusing on detail and the individual parts of the whole and associated difficulties in top-down processing, set shifting and disregarding unrelated details. For that reason, individuals with Autism have to work slowly so as to continue to keep a global view of what they are doing (Happe 2005; Shah and Frith 1993; Spek et al. 2008, 2017). Spek et al. (2017) also tried to unpick what contributed to the impairment in processing speed in older adults with Autism. By looking at their participants’ performance on the two processing speed subtests on WAIS-III, they found that older adults with Autism (aged 60 or above) only displayed impairment in one of the subtests, Digit symbol-coding (DSC) subtest, but not the Symbol Search (SS) subtest. However, the present study did not replicate such findings as we found control group outperformed Autism group on both PSI subtests. One possible reason as to why we did not replicate Speck et al. (2017)’s findings might be due to that fact that the average participant’s age in the current study was younger than those in Spek et al. (2017)’s study. In addition, the publisher of WAIS-IV identified the measurement of processing speed and working memory as two areas of weakness in WAIS-III. After incorporating Carroll’s Three Stratum Theory (Carroll 1993, 1997) as the theoretical blueprint for WAIS-IV (Taub and Benson 2013; Wechsler 2008c), several changes were made to WAIS-IV subtests to improve reliability and validity of the measurement of processing speed and working memory. For example, more symbols were added to the coding task and new trials of digit span forward and backwards were added (Taub and Benson 2013). In other words, the WAIS-IV subtests might be more sensitive in picking up difficulties in processing speed experienced by individuals with Autism than the WAIS-III. Hence, this might be another reason why the current study did not replicate Speck et al’s (2017) results.

Another potential explanation of poorer processing speed performance in older adults with Autism is that they have a tendency to adopt a more careful and conservative response strategy. This hypothesis is supported by a previous study (Lever et al. 2017) that looked at inference control in individuals with Autism. Lever et al. (2017) also found that middle-aged and older adults with Autism employed a more conservative response strategy than young adults with Autism who did not adopt the same accuracy over speed bias. Similarly, it is possible that older adults with Autism in the present study adopted the same strategy when they approached the processing speed subtests and thus leading to lower PSI scores compared to the controls.

### Memory Abilities

Older adults with Autism performed at a similar level to those without Autism on auditory memory, visual memory, immediate memory and delayed memory, but differed in visual working memory. A similar pattern of results was found for IQ adjusted memory scores, in which TD older adults outperformed older adults with Autism in visual working memory.

Previous findings suggests that age-related cognitive decline and working memory performance might be influenced by several factors, for instance decline in sensory functioning, poorer interference control and reduced processing speed (Salthouse 1996; Baltes and Lindenberger 1997; Hasher and Zacks 1988). Some of these factors are also known to be characteristics of Autism, therefore it is possible that working memory performance in older adults with Autism represents a ‘double jeopardy’ effect of Autism, whereby Autism may have an additional negative impact on age-related cognitive decline.

One interesting finding in our study is that older adults with Autism performed worse in visual working memory tasks than typically developed controls but no group difference was found in verbal working memory tasks. This difference between verbal and visual tasks may also explain some of the discrepancies in previous literature on working memory differences. For instance, Geurts and Vissers (2012) found deficits in visual working memory in older adults with Autism, whereas Lever et al. (2015) revealed similar level of working memory performance in individuals with Autism and TD controls. Geurts and Vissers (2012) used the Spatial Span task from WMS-III, whereas Lever et al. (2015) employed the N-back task. Unlike the stimuli used in the Spatial Span task, the N-back task uses simple pictures as stimuli that are easy to name or verbalise. It was therefore hypothesized that participants with Autism might have used verbal strategies, which could have helped them perform better (Koshino et al. 2005; Williams et al. 2005). Hence, individuals who are aged over 50 with Autism may have difficulties in working memory and may be limited to the processing of visual information that is not easily verbalised.

### Strengths and Limitations

Strengths of this study pertain to the use of a well-validated test battery measuring both cognitive and memory abilities. One of the reasons why there has been inconsistent findings regarding cognitive and memory abilities in older adults with Autism is the use of less robust and comprehensive measures. Therefore, the present study aimed to build on previous work by employing the WAIS-IV and WMS-IV, which have high reliability and validity in assessing intellectual abilities and various memory abilities. In addition, the present study is also the first to look at IQ adjusted memory abilities which allows more accurate interpretation of participants’ memory abilities as the influence of any IQ differences between individuals can be controlled for. Furthermore, all participants with Autism in the present study were aged of 50 or over and there have only been a handful of studies that explored cognitive functioning in individuals with Autism in this age group.

There are also several limitations to the present study. Participants were all in the normal to high functional range without any co-morbid intellectual disabilities in order to focus specifically on Autism without the confounding factor of having an intellectual disability. This might limit the representativeness of the present findings as there is a high prevalence rate of Autism in individuals who have co-occurring intellectual disabilities (Matson and Shoemaker 2009).

Secondly, although the National Autistic Society (2013) and previous research converged upon the idea that individuals with Autism aged 50 or above are considered as older adults, age-related cognitive decline in TD individuals is reported to generally have an onset at around 60 years of age (Nilsson et al. 2009; Treitz et al. 2007; Geurts and Vissers 2012). While the participants’ median age was 61 years old and there was a wide range of ages participating in the study, many individuals in the present study were under the age of 60. Therefore, it may be best to consider the current findings as pertaining to a ‘young old’ Autism group. Future studies should be conducted to check if they can replicate the present findings with an older sample (aged 60 or above). It is also worth noting that the present study was cross-sectional, rather than longitudinal, thus no firm conclusions can yet be drawn as to how cognition changes as individuals with Autism age. Nonetheless, the current sample provided some initial insight into the cognitive and memory abilities in individuals with Autism as they enter late adulthood.

Thirdly, the Autism and control group were not balanced in terms of gender with there being a higher proportion of female to male participants in the control group, and a reverse pattern was found in the Autism group. In the Autism group this is a pattern that tends to be seen in the relative proportion of males to females receiving a diagnosis. A recent systematic review revealed that the male to female ratio in meeting criteria for Autism is about 3:1 (Loomes et al. 2017). As mentioned in the results section, gender was found to be associated with processing speed performance. While our regression analyses suggested that gender did not confound the relationship between diagnosis and cognitive performance, future studies should continue to explore the effect of gender on cognitive performance with a larger sample of autistic older adults and TD older adults as previous studies have found that age-related decline begins earlier in TD men than in TD women and there is no significant gender difference in terms of prevalence of dementia in TD population (Ruitenberg et al. 2001; Gur and Gur 2001).

In addition, we recruited participants with Autism if they reported that they received a clinical diagnosis of Autism prior to the study. However, we did not employ other diagnostic measures, i.e. AQ and ADOS, to ascertain whether their Autism diagnoses were accurate. On reflection, the Autism Quotient (AQ) could have been given to participants with Autism to verify their diagnosis as the controls were asked to complete AQ prior to the experiment.

Another limitation is related to the design of the present study. On average, each participant took about 3.5 h to complete all the cognitive assessments and a fatigue effect might have presented as a result of long testing duration. However, attempts were made to mitigate any effect of fatigue by counterbalancing the order in which the neuropsychological assessments were administered.

### Clinical Implications and Future Research

By considering the cognitive and memory abilities of older adults with Autism, this study furthers our understanding of the levels of cognitive functioning in this population and it allows us to examine whether cognitive differences evidenced in children and adolescents with Autism persist into older adulthood.

Deficits in processing speed and working memory have previously been found to be evident in children and adults with Autism (Geurts et al. 2004, 2008; Zinke et al. 2010; Spek et al. 2008; Powell et al. 2017). The present findings indicated that these deficits seem to persist when this population enters late adulthood. Processing speed performance is of particular importance as it has been found to be closely related to levels of independence in daily functioning (Davids et al. 2016; Gothe et al. 2014; Marshall et al. 2011; Bell-McGinty et al. 2002; Jurado and Rosselli 2007), hence more research into processing speed could be of utility in promoting independent living in individuals with Autism. With better knowledge of cognitive functioning in older adults with Autism, the development of intervention and services for this population can be facilitated so as to meet their needs.

While the present study found evidence that older adults with Autism had poorer performance in visual working memory compared to their TD peers, no significant group difference was found in delayed memory, which has been found to be the most and earliest affected in Alzheimer’s disease (Salmon and Bondi 2009). This is to say our findings suggest that having an Autism diagnosis does not seem to confer a risk factor for Alzheimer’s disease, the most common type of dementia. However, our findings are cross-sectional and thus caution needs to be applied in relation to conclusions for a longitudinally deteriorating condition such as dementia.

To date, all studies, including the present study that investigated cognitive functioning in older adults with Autism have been cross-sectional in design and no longitudinal studies have yet been conducted to examine the cognitive changes in this population as they age. For that reason, longitudinal studies are warranted to explore how the combination of ageing and Autism affects cognitive functioning over time in individuals with Autism.
